# Supplementary material for: Monitoring of activity-driven trafficking of endogenous synaptic proteins through proximity labeling
Source: PLoS Biol. 2024 Oct 28;22(10):e3002860. doi: 10.1371/journal.pbio.3002860 (PMC11542813; doi:10.1371/journal.pbio.3002860)

Figure 1F

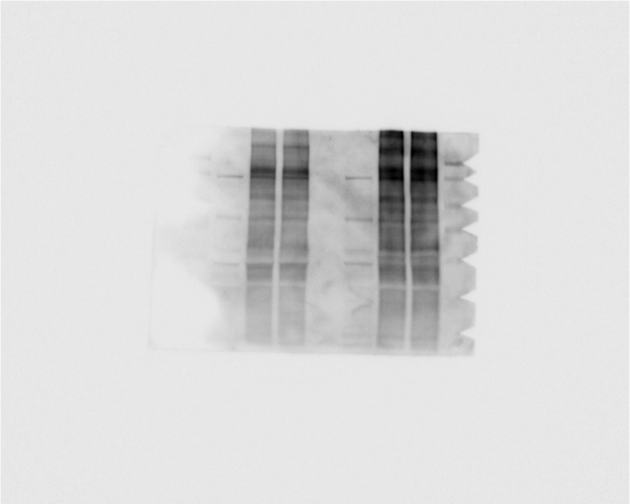

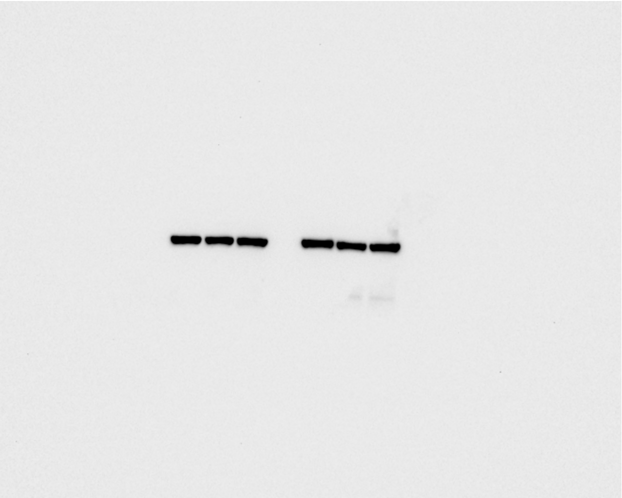

In the Fig 1F were used the three first lanes of the uncropped blots shown here.

Figure 2A

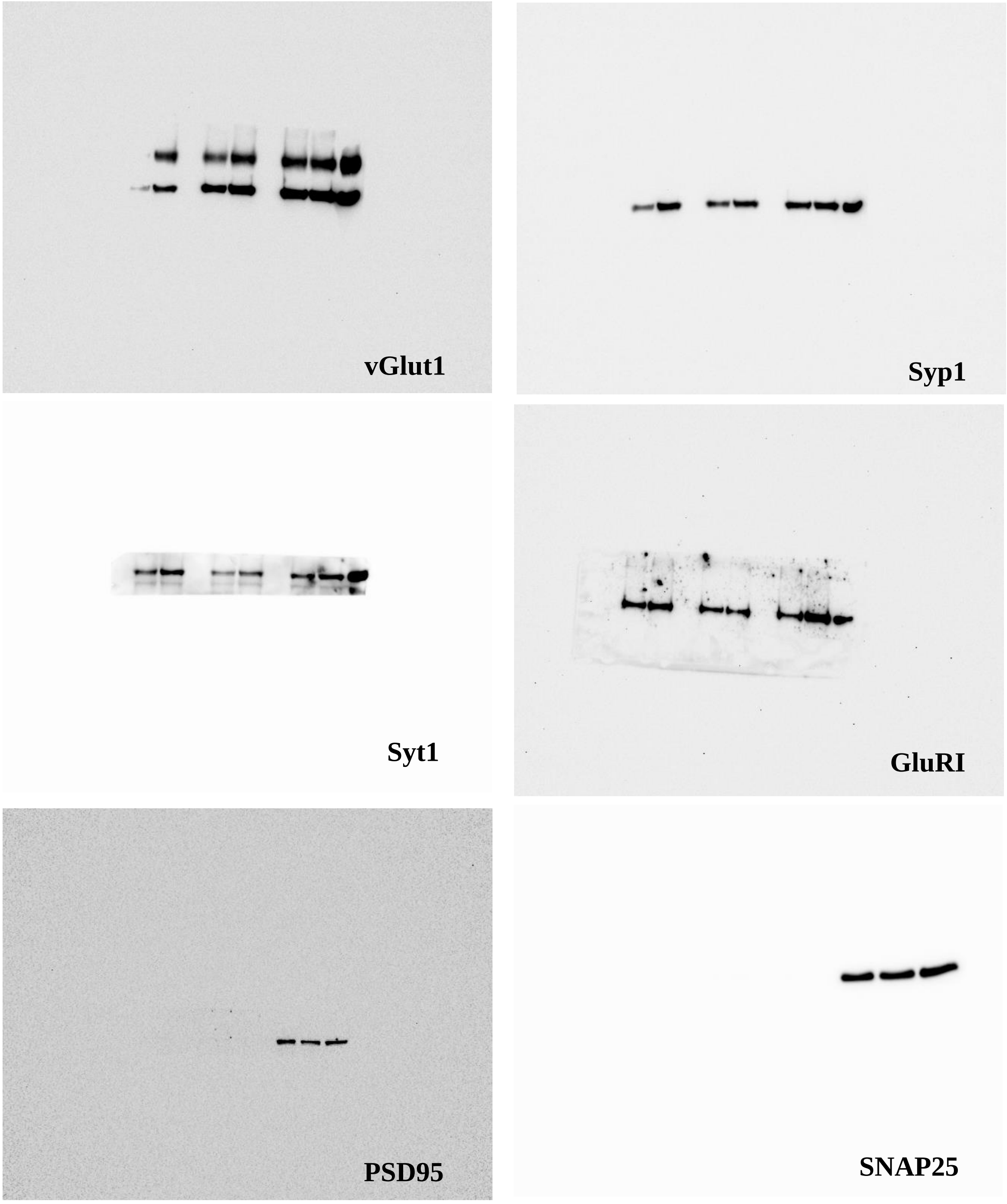

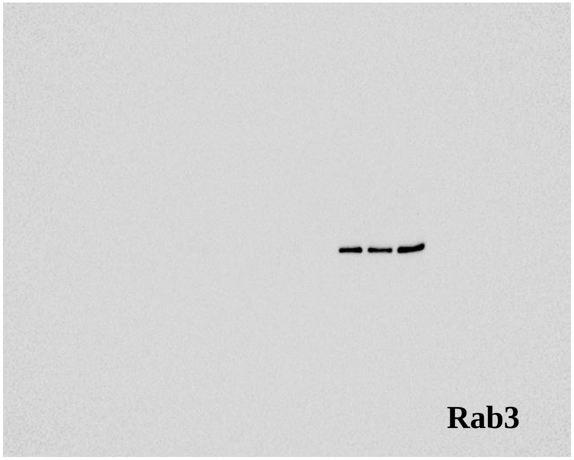

Three last lanes correspond to *Inputs* from total lysates (5, 5 and 8 μg protein from left to right).

Figure 2D

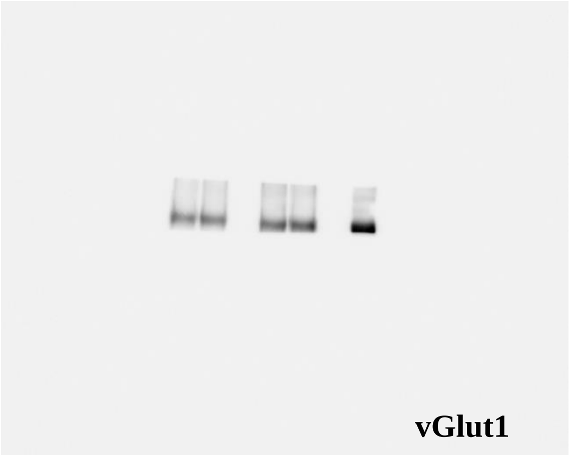

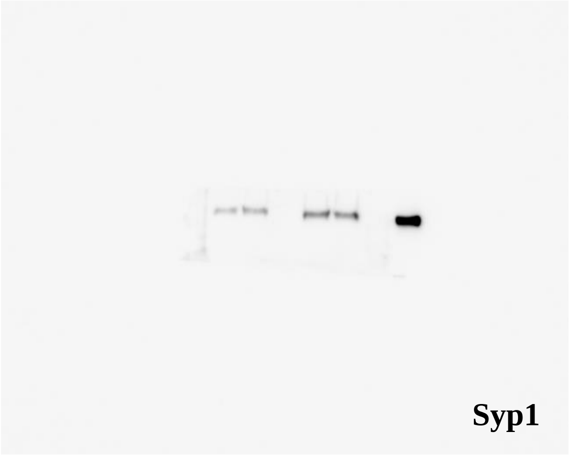

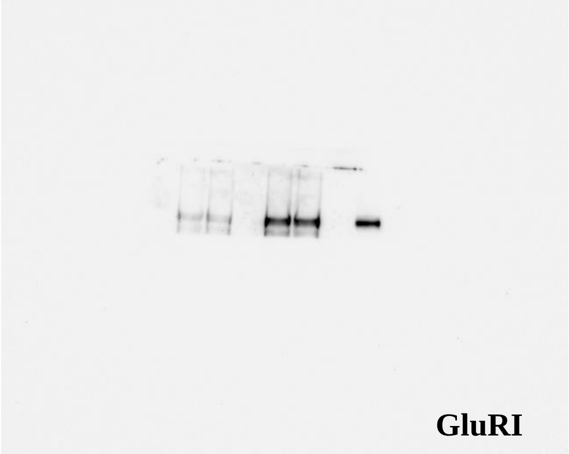

In the uncropped blots of Figs 2D, 3A and 4A, two first lanes correspond to another biological replicate (*Control and KCl*) of the same experimental approach.

Figure 3A

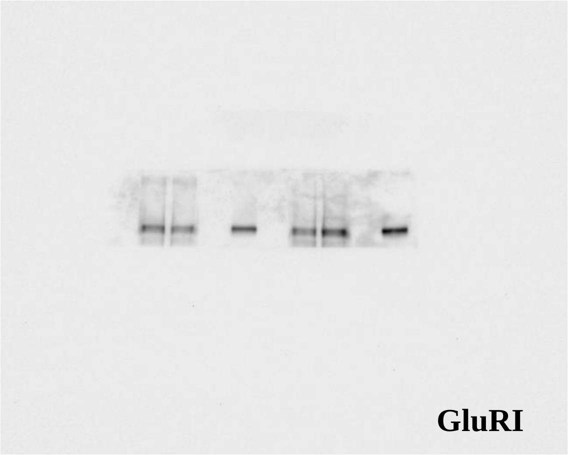

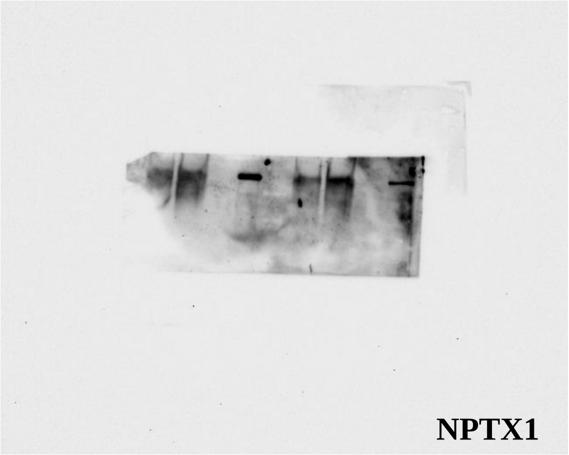

Figure 4A

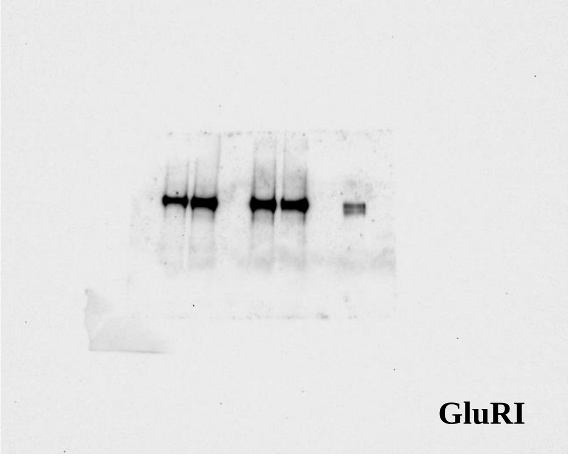

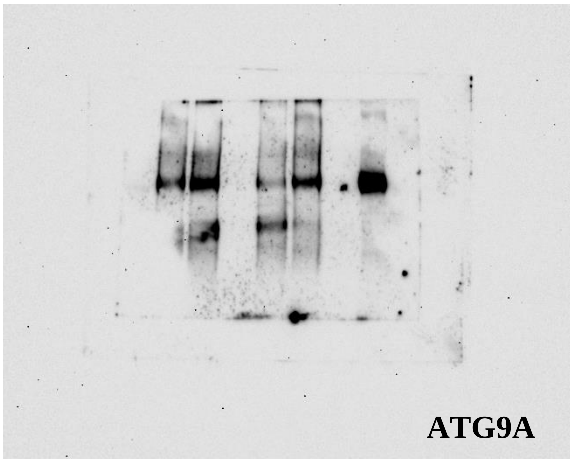

Supp. Figure S2

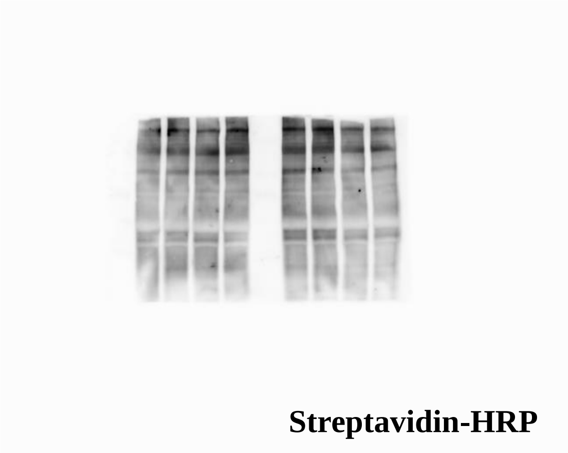

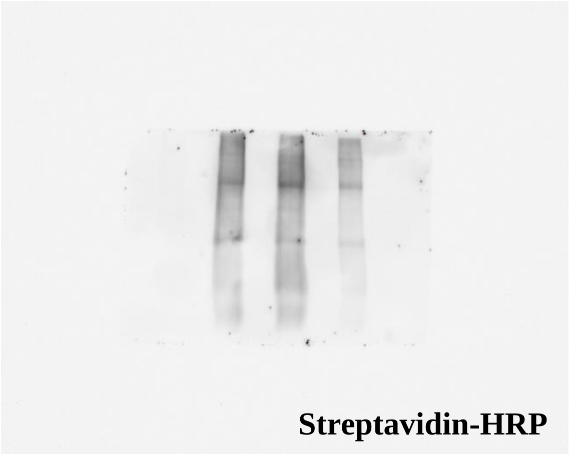

Supplement: S1 Raw Images — This file contains the uncropped chemiluminescence images for the western blot results represented in Figs 1F, 2A, 2D, 3A, 4A, S2A, and S2C. (PDF) [file pbio.3002860.s006.pdf]
