# Supplementary material for: Monitoring of activity-driven trafficking of endogenous synaptic proteins through proximity labeling
Source: PLoS Biol. 2024 Oct 28;22(10):e3002860. doi: 10.1371/journal.pbio.3002860 (PMC11542813; doi:10.1371/journal.pbio.3002860)
Supplement: S1 Protocol — This section contains the step by step detailed protocol for the preparation of neuronal embryonic cultures, synaptic cleft proximity biotinylation and lysate collection for some of the experiments carried out in this study. (DOCX) [file pbio.3002860.s007.docx]

## Extended supplementary protocol

Synaptic cleft proximity labeling during neuronal activity

**Summary:** This protocol details the steps for using proximity biotinylation to isolate synaptic cleft proteins in firing neurons and quantify their changes in synaptic surface abundance by western blot. We aim to provide detailed information to ensure reproducibility and highlight the steps we have found to be essential for consistent results. This protocol is strongly derived from previous work described in Hung et al.[^1^](https://www.zotero.org/google-docs/?broken=WYDJP2) and Loh et al.[^2^](https://www.zotero.org/google-docs/?broken=Mhy4tb), including the composition of most solutions used and steps required for consistency, but it presents several modifications essential for capturing trafficking events at the synapse. Details of media composition are described at the end of the protocol.

Procedure:

**A. Preparing neuronal cultures expressing LRRTM-HRP:**

**
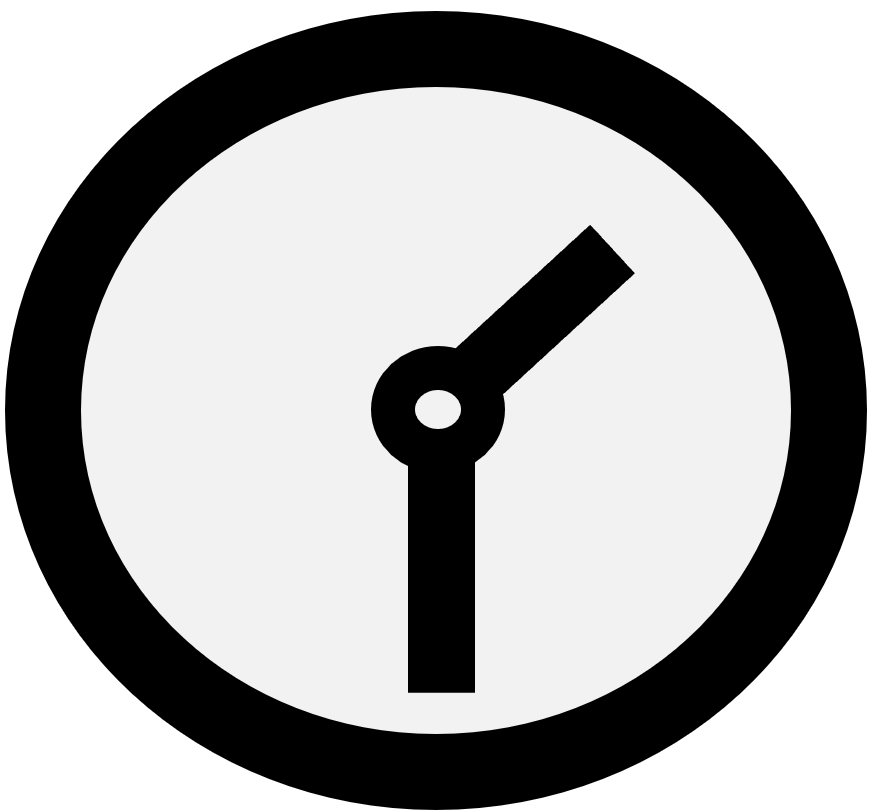
 TIMING: 7-8 h (distributed in different days)**

1. Coat 2 x 10 cm culture dishes per experimental condition with 0.1 mg/ml poly-D-lysine. Coat for 72 h before plating the cells, leaving the dishes with the coating solution in the incubator at 37°C for at least 48 h. Remove the coating solution and wash two times with deionized water 24 h before plating cells. Finally, dry the dishes in the incubator overnight.
2. Plate 3.5-4 million cells isolated from rat E18 cortex per 10 cm dish. Using this neuronal density ensures an optimal number of individualized neurons to obtain a good amount of protein and avoid the formation of neuronal clumps. Two dishes should provide around 1.4-1.8 mg of protein, which is an ideal amount to be able to run several separate western blots to quantify different proteins from the same original sample.
3. Plate neurons using *Plating media* and 4-5 days later replace this media with *Maintenance growth media*, replacing half of media every 3-4 days. Note that our version of *Maintenance growth media* contains BrainPhys medium, which in our experiments increased neuronal viability compared to the media used by Loh et al.^1^
4. At DIV15, transduce neuronal cultures with lentivirus to express both FSW-HRP-V5-LRRTM1 and FSW-HRP-V5-LRRTM2 in equal amounts. To do so, dilute ~3 x 10^8^ viral particles (measured by ELISA as detailed in the manuscript) of both constructs per 10 cm dish in 1 ml of fresh BrainPhys medium and drop this mix onto each 10 cm dish. Remember to thaw and keep lentiviruses on ice. Lentiviral aliquots can be frozen and used again at least one additional round.

**B. Surface proximity biotinylation and lysate collection.**

**TIMING: 5.5-6 h (variable depending on the number of dishes).**


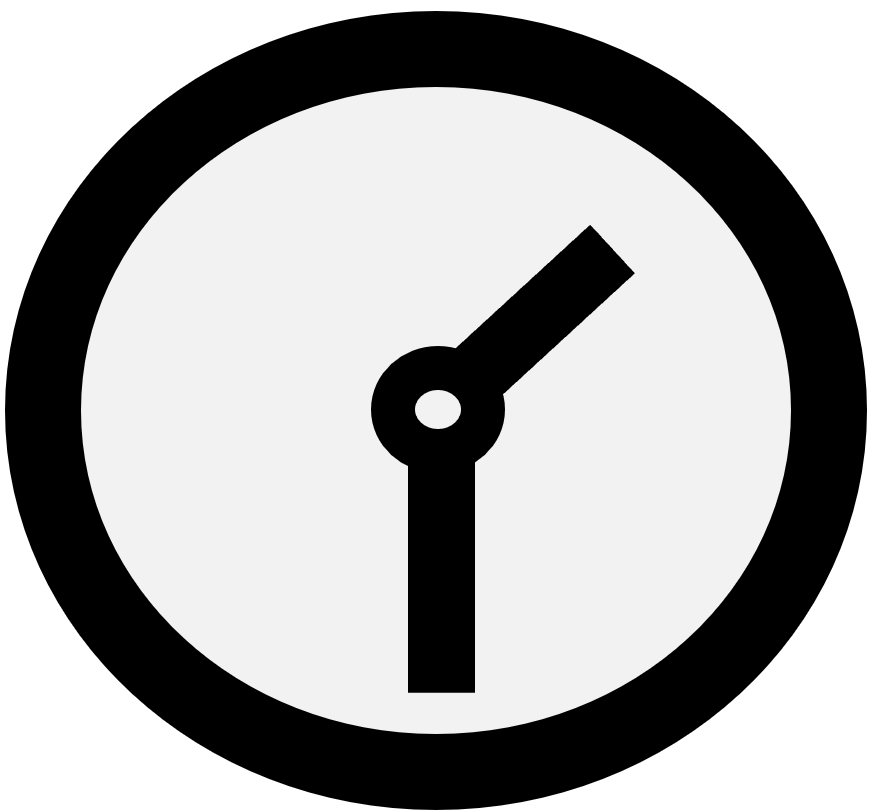


1. Start the synaptic cleft proximity biotinylation assay at DIV19. This provides sufficiently mature cultures presenting synapses and enough time for expression of transduced HRP-LRRTM constructs. Note that these experiments do not require a biotin starvation step before starting, as biotinylation occurs selectively in the extracellular space using impermeant BxxP biotin substrate and enriched surface localization of HRP enzymes.

*Tip: A well-organized setup is essential to perform the experiment in the required time frame.*

1. Make sure to have solutions prepared in advance before starting the protocol, including Tyrode’s solutions warmed at 37°C (Control, High KCl and quencher), 1X DPBS warmed at 37°C and cold 1X DPBS.

*Tip: Before starting, prepare solutions to trigger biotinylation reactions with the different conditions to be tested. For comparing neurons at rest to neurons activated with KCl, prepare individual tubes with the solutions and the exact amount of volume needed so you can simply drop the solution into the corresponding dish. For example, prepare control or 90 mM KCl Tyrode’s solutions supplemented with 100 μM BxxP impermeant-biotin phenol and 1 mM H_2_O_2_ and then distribute 8 mL in 15 mL tube in as many tubes as dishes you have planned for the experiment. At least 8 mL of volume are needed to completely cover the surface of a 10 cm dish.*

1. Have the CultureTemp Warming Plate (or similar surface heating platform) connected at 37°C and prepare a large recipient with ice for scraping cells.
2. The Tyrode's solutions must be prepared fresh for each experimental day. In the case of 90 mM KCl Tyrode’s, firstly we tested its efficiency by live imaging with an experiment similar to the one described in Fig 1E

*❕* ***Critical:*** *Prepare solutions fresh before each experiment.*

1. Even though you use 2 dishes per experimental condition (as in our case), you should perform the experiment one by one in order to strictly respect the 15 second reaction time. The sequence of steps to proceed in each experiment would be as follows:

- Take a dish from the incubator and put it on the CultureTemp Warming Plate.
- Aspirate culture medium and wash with warmed 1X DPBS

*❕* ***Critical:*** *do not skip this step, this wash will remove dead neurons, any debris or undesired substances in the culture medium.*

- Aspirate the 1X DPBS solution and add 8 mL of control or 90 mM Tyrode’s solution for 15 seconds (or alternative time of reaction to be tested).
- Remove and discard the Tyrode’s solution and replace it by the quencher solution quickly, which will stop the biotinylation reaction.

*❕* ***Critical:*** *ensure very carefully the exact time of the biotinylating reaction. Aim to exchange buffers quickly so as to reduce the noise arising from inexact reaction times. Both starting the reaction and stopping it should be done quickly.*

- Wash with the quencher solution two times more, taking around 5 seconds per wash.

*❕* ***Critical:*** *Be sure to remove well the washing solutions in each washing step to remove possible contaminants.*

- Carefully aspirate the rest of the solution left after the last washing.
- Wash once with cold 1X DPBS, aspirate and place the dish on the ice tray. This dish contains neurons in which the synaptic cleft has been labeled through proximity biotinylation.
- Scrape the cells with 400 μL of cold 1X DPBS and keep the cell suspension in a 1.5 mL tube in ice.

*Tip: Save at this point 20-30 μL of the DPBS scrapping mix containing the neurons for protein estimation using the bicinchoninic acid (BCA) assay for protein quantification. It is important to do so now, as the presence of ascorbate and Trolox in the lysis buffer solution used in next steps makes protein estimates less reliable.*

1. Centrifuge the remaining DPBS scrapping mix containing the neurons at 3,000 g for 10 minutes at 4°C to pellet the cells.
2. Remove and discard the supernatant, and resuspend the pellet containing the sample of interest in 100 μL of 1% SDS lysis buffer as indicated in Loh et al.^1^. This will require continuous yet mild homogenization.

*Tip: To facilitate this resuspension, it is recommended to cut the end of a regular 200 μL tip, as the first pipetting can be tedious.*

1. Boil samples for 5 min at 95°C with gentle shaking. This can be easily achieved using a ThermoMixer C (Eppendorf, 5382) in which shaking is set at 350 rpm.
2. Next, add 400 μL of 1.25X RIPA Buffer per sample and mix gently. After the combination of 1% SDS and 1.25X RIPA solutions, the cell pellets are considered lysed and diluted in 1X RIPA lysis buffer. *Note: If you use a higher volume of 1% SDS lysis buffer to dissolve the pellet in step 11, then you must also adjust the volume of 1.25X RIPA buffer to add for finally getting your lysates in 1X RIPA lysis buffer.*

*❕* ***Critical:*** *Steps 13 and 14 are essential to disrupt the strong interactions occurring within postsynaptic densities, as previously demonstrated by Loh et al^1^. This step is essential to favor the isolation of synaptic cleft proteins without undesired cytosolic proteins. A good control can be to blot against PSD95 to ensure that it is not isolated through interactions with surface proteins at the postsynapse.*

1. Incubate the lysates for 30 min at 4°C in gentle rotation using a Tube Revolver.

*Tip: use this time to measure protein concentration of samples from step 9.*

1. Clear the lysates by centrifugation at 16,000 g for 10 min at 4°C. This step will pellet undesired unlysed structures. Recover the supernatant, which will contain a mix of proteins containing both biotinylated and non-biotinylated proteins.

*Tip: At this point the experiment can be paused. If so, lysates must be stored at -80°C. They can be stored for several months.*

**C. Streptavidin Enrichment and Characterization by Western Blotting.**

**TIMING: 1h overnight incubation + 2.5h (until running the SDS-PAGE gel).**


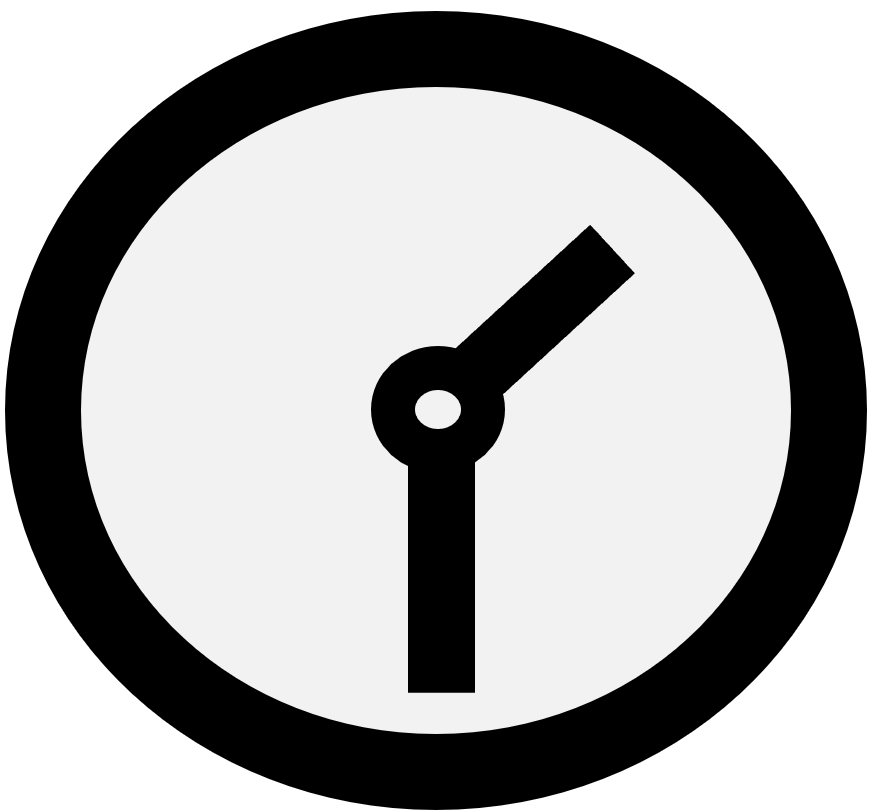


1. Synaptic cleft proteins now have to be enriched from the lysates using Streptavidin Magnetic Beads. Each protein of interest to be studied may require different amounts of initial sample for optimal detection by western blot. As a reference, synaptic vesicle proteins such as vGlut, Synaptophysin 1 or Synaptotagmin 1 are detected clearly when using 100 μg of protein lysate together with 30 μL of Streptavidin Magnetic Beads slurry.

*Tip: There are three main factors to take into account when detecting proteins in the synaptic cleft by western blot. Detection will be favored by 1) high abundance at the synaptic cleft, 2) large activity-driven translocation to the synaptic surface and 3) specific and high-affinity of the antibody used for detection. If these three conditions are present, 100 μg of protein lysate should suffice for detection. When any of these conditions was not met, we increased the initial sample of lysate to 200–300 μg of protein and 60-90 μL of Streptavidin Magnetic Beads slurry. Optimization is likely necessary for proteins not described in our work.*

1. Wash Streptavidin Magnetic Beads two times with 1 mL RIPA lysis buffer.
2. Incubate washed beads with lysate at 4°C overnight with gentle rotation.

*Tip: To facilitate the correct incubation, the beads must be incubated within a total volume of at least 750-800 μL. If the required cell lysate does not fill to this volume it must be adjusted with 1X RIPA lysis buffer.*

1. The next day the Streptavidin Magnetic Beads will require a series of 1 minute cold washes as follows:

a) 2 washes with 1 mL RIPA lysis buffer

b) 1 wash with 1 mL of 1M KCl, 1 mL of 0.1 M Na_2_CO_3_, 1 mL of 2 M urea

c) 2 washes again with 1 mL RIPA lysis buffer

d) 1 wash with 1X DPBS

*Tip: Each wash is carried out for 1 min through gentle rotation using a Tube Revolver. All buffers are freshly prepared on the day of the experiment and used at 4°C.*

1. After the last wash, elute biotinylated proteins from the beads by adding 25 μL of 3X protein loading buffer (supplemented with 20 mM DTT and 2 mM Biotin) per sample and boiling for 10 min at 95°C with agitation to favor the elution (550 rpm in ThermoMixer C).
2. Finally, the streptavidin eluate is collected using a DynaMag -2 and run on an SDS-PAGE gel.

*Tip #1: Tip: Adjust the percentage of acrylamide depending on the size of your protein of interest. In our case, we used an 8% gel for larger proteins and a 10% gel for smaller proteins.*

*Tip #2: To ensure that you do not lose any biotinylated protein after elution, you can also load the beads into the gel together with the eluted sample. To do this, you can leave running the gel until the sample is partially packaged, stop the run, recover the beads with 10 μL of 1X LDS-DTT and load the beads into the corresponding wells).*

**D. Composition and storage of buffers and solutions used.**

Most of the solutions and compounds used in this experiment were strongly based on the work described by Hung et al.^2^ and Loh et al.^1^.

**Coating solution:** 1 mL of 10 mg/mL poly-D-lysine (previously prepared in deionized water) was dissolved in 100 mL UltraPure Distilled Water to give 0.1 mg/mL final concentration. This solution was prepared fresh the day of coating, but it can be stored at 4°C.

**Plating Media:** 50% of MEM supplemented with 2 mM L-glutamine, 10% FBS, 2% N21 + 50% of Neurobasal medium supplemented with 2% N-21 and 1% GlutaMAX. Stored at 4°C.

**Maintenance growth media:** BrainPhys Neuronal Medium supplemented with 2% SM1, 12.5 mM Glucose and 10 μM FUDR. Stored at 4°C.

**Control Tyrode’s solution:** 142.25 mM NaCl, 4 mM CaCl_2_, 3 mM KCl, 1.25 mM MgCl_2_, 0.5 mM NaH_2_PO_4_, 10 mM Glucose, 10 mM HEPES, 10 μM CNQX and 50 μM AP5 in UltraPure Distilled Water. *Buffered to pH 7.4 at 37°C.* Prepared fresh the day of the experiment and stored at 4°C until used.

**High KCl Tyrode’s solution:** 55.25 mM NaCl, 4 mM CaCl_2_, 90 mM KCl, 1.25 mM MgCl_2_, 0.5 mM NaH_2_PO_4_, 10 mM Glucose, 10 mM HEPES, 10 μM CNQX and 50 μM AP5 in UltraPure Distilled Water.

*Buffered to pH 7.4 at 37°C.* Prepared fresh the day of the experiment and stored at 4°C until used.

**Quencher Tyrode’s solution:** 142.25 mM NaCl, 1.25 mM CaCl_2_, 3 mM KCl, 1.25 mM MgCl_2_, 0.5 mM NaH_2_PO_4_, 10 mM Glucose, 10 mM HEPES, 10 μM CNQX and 50 μM AP5 in UltraPure Distilled Water. *Buffered to pH 7.4 at 37°C.* Prepared fresh the day of the experiment and stored at 4°C until used.

This solution was supplemented with 10 mM sodium azide, 10 mM sodium L-ascorbate and 5 mM Trolox before performing the experiment.

**1 M Sodium azide:** powder was dissolved in deionized water and stock stored at -20°C.

**1 M Sodium L-ascorbate:** powder was dissolved in deionized water the day of the experiment and stored at 4°C until used.

**500 mM Trolox:** prepared in DMSO the day of the experiment and kept at RT until its use. It can be sticky when handled cold, so remove several minutes before from the refrigerator to temper.

**BxxP impermeant-biotin phenol:** 100 mM stock prepared in DMSO and stored at -20°C.

*Note: In the original work from the Ting lab, BxxP was custom-synthetized, but now it is commercially available from ApexBio Technology (Reference A8012).*

**1 M H_2_O_2_:** stock prepared the day of the experiment by dilution in 1X DPBS from 9.68 M 30% (w/w) commercial solution (stored at 4°C).

**1% SDS lysis buffer:** Sodium dodecyl sulfate powder was dissolved in 50 mM Tris-HCl (pH=8.0) and supplemented with 1X protease inhibitor cocktail, 1 mM PMSF, 10 mM sodium azide, 10 mM sodium L-ascorbate and 5 mM Trolox. Stored at 4°C.

**1X RIPA lysis buffer:** 150 mM NaCl, 0.1% SDS, 0.5% sodium deoxycholate, 1% Triton X-100 prepared in 50 mM Tris-HCl (pH=8.0). Stored at 4°C.

**1.25X RIPA lysis buffer:** 150 mM NaCl, 0.2% SDS, 0.5% sodium deoxycholate, 1% Triton X-100 prepared in 50 mM Tris-HCl (pH=8.0). Stored at 4°C.

**1 M KCl:** powder was dissolved in deionized water and stored at 4°C.

**0.1 M Na_2_CO_3_:** Na_2_CO_3_ was dissolved in deionized water and stored at 4°C.

**2 M Urea:** 8 M Urea from commercial stock was diluted in 10 mM Tris-HCl solution (pH=8.0) the day of the experiment.

**3X protein loading buffer for elution biotinylated proteins:** LDS-PAGE 4X was combined with UltraPure Distilled Water in a volume ratio 3:1 and supplemented with 20 mM DTT (from 1 M stock prepared in deionized water) and 2 mM Biotin (from 100 mM stock prepared in DMSO). Stored at -20°C.

**10X Tris-Buffered Saline (TBS):** 0.5 M Trizma base (Merck, T1503) and 1.5 M NaCl prepared in deionized water. To adjust pH to 7.5 with HCl. Stored at RT.

**TBS-0.2% Tween:** 0.2% Tween-20 (Merck, P1379) dissolved in 1X TBS (prepared in deionized water from 10X TBS). Stored at RT.

**10X SDS Running Buffer:** 1% SDS, 0.25 M Trizma base and 1.92 M Glycine (Merck, G8898) prepared in deionized water and stored at RT. The 1X solution used for running SDS-PAGE gels was also diluted in deionized water.

**10X Transfer Buffer:** 0.25 M Trizma base and 1.92 M Glycine dissolved in deionized water and stored at RT. Expected range pH= 8.4-8.7. For transferring protein gels to nitrocellulose membranes this buffer was used 1X in a solution composed of 70% deionized water, 20% Methanol (Merck, 32213) and 10% 10X Transfer buffer.

**E. Final notes on primary cultures of cortical neurons.**

We obtained consistent and reproducible results using cortical neurons obtained from E18 rat embryos. We initially compared the use of 5-Fluoro-2'-deoxyuridine (FUDR) and Cytosine Arabinoside (Ara-C) for reducing the presence of astrocytes in the cultures, finding that FUDR provided a much higher viability of cultures and consistently enabled us to maintain healthy neurons up to DIV19 when the experiment was performed. We also explored the use of co-cultures of astrocytes and neurons as the initial sample for synaptic cleft isolation. These were obtained from postnatal rat pups (day 0-2), as this is a sample that in our hands has been ideal for imaging studies. However, we found that this sample did not enable reproducible results on visualizing activity-driven trafficking of endogenous synaptic proteins, so we switched to embryonic rat cultures (E18). We also observed that the expression of neuronal markers per microgram of protein was higher in embryonic cultures than in postnatal cultures. We reasoned that having exclusively neurons and not astrocytes likely facilitates the isolation of synaptic neuronal proteins, increasing reproducibility in our hands. Future work will be needed to optimize the use of co-cultures for these experiments.


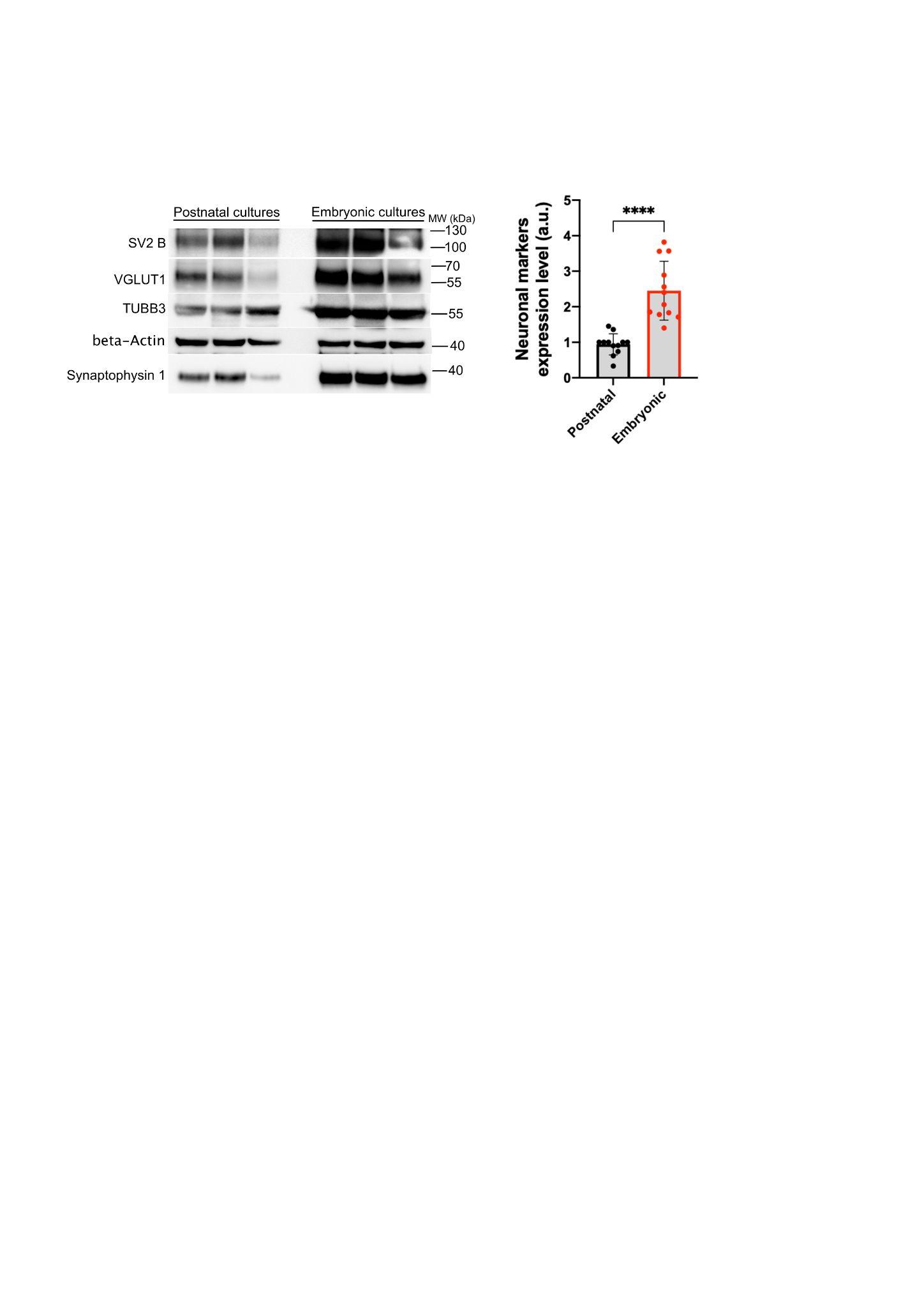


***Extended protocol Figure 1. Comparison of neuronal markers in cultures of neurons or co-cultures of neurons and astrocytes.*** *Left: Representative western blot from whole lysates of rat postnatal and embryonic cultures at DIV18 using the same amount of protein loaded. Western blots were developed against different neuronal markers: synaptic vesicle glycoprotein 2B (SV2B), vesicular glutamate transporter 1 (VGLUT1), synaptophysin1 (Syp1) and neuronal marker beta-Tubulin III (TUBB3); beta-Actin protein was established as loading control. Right: Quantification of neuronal marker expression (from three different biological replicates) in postnatal and embryonic cultures normalized against beta-Actin expression. Unpaired t-test, **** p<0.0001.*

**F. References.**

1. Loh, K.H., Stawski, P.S., Draycott, A.S., Udeshi, N.D., Lehrman, E.K., Wilton, D.K., Svinkina, T., Deerinck, T.J., Ellisman, M.H., Stevens, B., et al. (2016). Proteomic Analysis of Unbounded Cellular Compartments: Synaptic Clefts. Cell *166*, 1295-1307.e21. https://doi.org/10.1016/j.cell.2016.07.041.

2. Hung, V., Udeshi, N.D., Lam, S.S., Loh, K.H., Cox, K.J., Pedram, K., Carr, S.A., and Ting, A.Y. (2016). Spatially resolved proteomic mapping in living cells with the engineered peroxidase APEX2. Nature protocols *11*, 456–475. https://doi.org/10.1038/nprot.2016.018.
