# Supplementary material for: Monitoring of activity-driven trafficking of endogenous synaptic proteins through proximity labeling
Source: PLoS Biol. 2024 Oct 28;22(10):e3002860. doi: 10.1371/journal.pbio.3002860 (PMC11542813; doi:10.1371/journal.pbio.3002860)
Supplement: Attachment — Submitted filename: PLoS Biology revision comments.docx [file pbio.3002860.s008.docx]

We thank reviewers for their constructive criticism, the time spent to revise our manuscript and the helpful comments that have made the new version clearly better than the initial manuscript. The point-by-point response to the reviewers and explanations related to their comments are listed below:

**Reviewer #1:** The goal of the authors is to develop a method that identifies the activity-driven synaptic cleft proteome, an important and outstanding question in the field. This work builds on a previously developed tool that localized the biotinylating agent to the postsynaptic membrane, but that, as used, could not identify the proteins that are transiently localized to the cleft during activity-driven SV exocytosis. Through spatiotemporal coincidence of neuronal activity and biotinylation, the authors show that they can detect proteins that could not be detected with the existing approaches. They validate the method by looking at two well-known synaptic vesicle proteins, and then extent their technique to demonstrate that ATG9 and NPTX1, two proteins that have been hypothesized to be present at the cleft during activity-driven exocytosis, could be detect through the newly developed biotinylation assays and through traditional pHluorin tagging. The technique is of value and beyond the technique, the studies on ATG9 and NPTX1 are of value to the field. We suggest additional controls and minor edits to improve the paper.

**Major revisions:**

1. The results for one of these two positive controls, VGlut1, is confusing. Can the authors comment on the high levels of vGlut1 in the Control lane under the 30s condition? Is this consistently observed? To that avail, could there be additional positive controls, like synaptotagmin proteins, that might strengthen these control observations?

Previous estimates indeed show that only a small fraction of vGlut is located on the neuronal surface (~3%; Kim and Ryan, 2009, J Neurosci). The reason we can detect such a small fraction (particularly under 30 seconds of biotinylation) compared to the input is that the input represents a much smaller amount of protein versus the amount of protein from which the enrichment is performed. In this case, the input line corresponds to 5% (5 µg) of the amount of protein used for surface isolation (100 µg). These conditions strongly favor the detection of enriched samples versus the input if labeling is efficient. Thus, despite only 3 out of 100 vGlut molecules potentially being detected from the surface, we magnify their detection in the western blot by using 20 times more protein as the initial sample for isolation compared to the input. This, together with increased biotinylation occurring during 30 seconds, reveals the small amount that is isolated, now detectable.

To clarify this important point, we now 1) show how much the input corresponds to in comparison to the sample used for isolation throughout all the figures of the manuscript, 2) add in figure legends the amounts of protein used in each case and 3) mention an explanation in the results section to clarify this point (see page 4, second paragraph).

We also have performed new experiments to evaluate whether our method can detect other proteins, such as Synaptotagmin 1, as suggested. New experiments shown in Figure 2A, B report a clear trafficking of endogenous Synaptotagmin 1 when neurons are stimulated for 15 seconds, supporting our conclusions. We have updated the text to comment on these new results (page 4, second paragraph).

2. It is not clear how the normalizations were performed for Fig 2B, C and similar graphs. Is it well known (and expected) that GluR1 does not vary due to activity at the synapse?

We aimed to control for biotinylation rates at the cleft in each experiment and found that the abundance of GluRI remained unchanged during activity (Figure 2C), providing a reliable control with a high-quality antibody. To our knowledge, it was previously unknown whether KCl would drive a short-term (i.e., 15 seconds) change in GluRI abundance at the synaptic cleft. However, we consistently find that cleft GluRI remains unchanged during KCl application. Thus, we considered it an appropriate control to quantify biotinylation rates at the cleft in each experiment. While Figure 2B is normalized to the detection of GluRI at the cleft in each particular measurement, Figure 2C only provides the ratio of change in GluRI abundance (and thus is not normalized to any other protein). This data is shown to establish the appropriateness of GluRI as a control for normalization. To clarify this point, we added additional explanations in the figure legend of Figure 2 (Page 24) and in the main manuscript (Page 4, third paragraph).

3. What explains the higher variability observed with the 15s KCl incubation? In the supplement it would be ideal to add curves of the mean+error bars of all the experiments that appear in 1C. In Figure 2B-C, the spread is also high in 15s, higher than that observed in 30s conditions. In general, it would be beneficial for the authors to comment on this variability observed for the technique, and how the variability observed for other biotinylation approaches.

We added individual curves, now shown in Supp. Figure S1B, and performed a few additional experiments to reinforce this point. There is indeed variability, likely arising from biological and experimental factors. In the case of variability in proximity labeling experiments with short biotinylation reactions, there is the additional complexity of exchanging buffers quickly enough to trigger and stop the reaction. The impact of experimental variability, such as spending 1 vs. 2 seconds exchanging buffers, is larger when the overall reaction time is shorter, which may contribute to the observed variability. Nevertheless, while quantitatively variable, the results consistently show activity-driven translocation. However, we don't think this variability can be compared to other biotinylation approaches (i.e., using external biotin sulfo-NHS), as labeling times in those approaches are 30 minutes and cannot be used for capturing fast activity-driven trafficking.

We added a phrase in the results section to comment on variability, as suggested (Page 4, third paragraph).

4. Because the temporal aspect of the biotinylation and activation are so crucial for this approach, in the results, the authors should briefly explain how to controlling biotinylation times, and extend this part o the discussion to future developments that would help better identify the activity-driven synaptic cleft proteome.

We now include a supplementary detailed protocol that provides experimental tips and suggestions on how to control biotinylation times, making it easier to reproduce our method. This should enable future developments in the use of this technique by the field. Additionally, we added a phrase in the discussion about the potential for scaling up this technique to resolve the activity-driven synaptic cleft proteome in the future, as suggested (Page 8, second paragraph).

Minor revisions:

5. Provide an explanation as to why imaging experiments were done in postnatal cortical neurons while the biotinylation assay was done in embryonic ones.

We have historically found that imaging experiments are more robust if astrocytes are present, enabling longer neuronal recordings. When developing our biotinylation strategy to capture activity-driven trafficking of endogenous proteins, we initially compared both embryonic and postnatal preparations and found much higher reproducibility with embryonic cultures. Additionally, if aiming to obtain a biochemical isolation as pure as possible, eliminating astrocytes from the preparation reduces the potential for nonspecific binding of unwanted astrocyte proteins to the beads. This will be helpful for the future establishment of this technique for mass spectrometry. As suggested, in our new supplementary detailed method we now add a short phrase mentioning that, in our hands, using postnatal co-cultures was not successful. We emphasize that the use of co-cultures is not reliable with the presented protocol and current set of tools, but that it may be achievable with appropriate modifications. We added a figure in the supplementary detailed protocol that shows that for a fixed amount of protein loaded in a gel, one finds higher levels of neuronal synaptic proteins in samples from embryonic cultures.

6. Where they Reframe Figure S1A - it is not clear what each square represents and it is not explained in the methods which datasets they used to create this figure or how they did it. What do they mean by "too few SV genes"?

We have expanded and clarified the section “Ontology analysis using SynGO”, in which we described this analysis originally (see page 18, first paragraph). As we now indicate in methods, each square of the figure represents a possible “subcellular localization” in synapses, which were pre-defined by the SynGO, an international consortium that aims to define the gene ontology of the synapse (www.syngoportal.org); “too few genes” indicates that no sufficient genes were identified in the original synaptic cleft proteome to belong to categories of subcellular localization belonging to synaptic vesicles defined by SynGO.

7. Figure 1A might benefit from having a legend that defines what each color or shape represents.

Fixed.

8. All plots that have error bars should specify which type of error bars they are. SD or SEM?

All error bars shown throughout the text represent SEM. This is noted in the Supplementary Table 1, which contains all details of statistical analysis per experiment.

9. In Figure 1C, what does each dot represent? Additionally, please place the statistical comparisons closer to the pair of groups that are being compared. Did the authors do a comparison between 15 and 30 seconds?

In Figure 1C each dot represents the response from a single neuron, in which we calculate from the total time in KCl, how much time the pHluorin signal is rising. We have clarified the statistical comparisons in the figure as suggested, and added a new comparison between 15s and 30s in the statistical tests. New results are also updated in the Supp. Table 1 containing details of statistical analysis.

**Reviewer #2:** The manuscript "Activity-driven trafficking of endogenous synaptic proteins through proximity labeling" developed an approach using synaptic cleft proximity labeling to capture and quantify activity driven trafficking of endogenous synaptic proteins at the synapse. The manuscript claimed that by carefully coordinating biotinylation and depolarization at synaptic clefts, and reducing labeling times to limit nonspecific labeling, proximity labeling enables the specific isolation and quantification of endogenous translocated proteins to the synaptic surface during neuronal activity. The main claim of the manuscript suggests the potential of this approach in providing direct evidence of the surface translocation of non-canonical trafficking proteins, such as ATG9A and NPTX1. The study adds to growing evidence that proximity labeling has emerged as one of the most useful research technologies to explore the trafficking of endogenous non-canonical proteins at the presynapse. The manuscript claimed that this strategy can be used to capture the translocation of any possible endogenous synaptic protein during neuronal activity. Further study demonstrated that endogenous NPTX1 and ATG9A are translocated to the synaptic surface, providing important biological insight into the functioning of these proteins at the synapses.

However, I have several major concerns that it needs more solid evidence to prove the author's viewpoint for publication. While the evidence that the approach enables capturing endogenous synaptic proteins involving into activity-driven trafficking through proximity labeling, the efficiency of this approach as well as the advantage of this approach need to be determined. It was also determined whether this approach can be used to identify new endogenous synaptic proteins in the synaptic surface during neuronal activity. Although this is potentially an important approach emphasizing the application of proximity labeling in the study of endogenous synaptic proteins, several data sets are preliminary.

Notwithstanding the above, I do have some comments/suggestions for the author's consideration. As listed below.

**Major revisions:**

1. The authors claimed that this approach facilitates directly assessing which certain proteins, hypothesized to participate in activity-driven translocation at synapses, indeed engage in such a process. However, the existed pHluorin-tagged constructs for these proteins also can enable such purpose to be achieved. From Figure 4F-4H, pHluorin-tagged constructs showed stronger signal intensity than the authors' approach. For example, pHluorin-vGlut1 showed a ~6-fold increase in the number of molecules found in surface abundance after field stimulation of 200 AP at 50 Hz, whereas vGlut1 showed only ca 3-fold increase in western blot of the isolation of synaptic cleft in the presence of KCl. pHluorin imaging methods also display advantage for other trafficking proteins, such as Syp1 and ATG9A. The authors should perform two methods, pHluorin imaging and synaptic cleft isolation, in same experimental conditions. Also, the authors did explain why field stimulation, but not KCL, was used to stimulate cells.

The essential concept to take into account when drawing the comparison between pHluorin and proximity labeling is that proximity labeling enables detecting the translocation of endogenous proteins. This is not the case when using pHluorin, which requires overexpression of pHluorin-tagged constructs. The central aim in our work is in fact to solve this long-standing issue by developing a strategy that enables quantifying synaptic translocation of any endogenous protein, surpassing the need to 1) modify the protein structure by adding pHluorin, a tag of significant size, and 2) overexpress the protein.

While a quantitative comparison shows better detection using pHluorin (a very established technique), our approach provides a unique value in demonstrating the translocation of endogenous proteins in their native state. To clarify this point, we added the word “endogenous” in a several parts of the manuscript to highlight the importance of this point (see Pag. 5, title section, middle and last paragraphs of the section; see Pag. 6, second and third paragraph; see Pag. 8, second paragraph).

In terms of quantitative comparisons, the very different nature of both techniques will inevitably alter the relative quantitative changes in surface translocation detected. Specifically: 1) pHluorin imaging is done in single neurons while proximity labeling reports populations of synapses from millions of neurons; 2) pHluorin constructs are overexpressed, while proximity labeling studies endogenous proteins expressed at their normal levels; 3) pHluorin results are obtained through imaging, while proximity labeling requires a series of intermediate processing steps that inherently increase the noise of the measurements. Our comparison in Figure 4 aims to show that, within the limits of each technique, similar results can be found when performing relative comparisons of protein translocation. This comparison is not expected to report similar surface fraction changes (due to different stimuli and techniques), but aims to demonstrate that proximity labeling can replicate the ballpark changes quantified using the gold standard in the field, pHluorin imaging.

We have in any case performed the suggested experiments and now show comparisons of pHluorin responses and synaptic cleft isolation using 15 seconds of KCl application. This new figure is shown in the new Supp. Figure S3G. These experiments include an additional protein, Synaptotagmin 1, to further support our conclusions. As expected, even with the same stimuli of KCl 15 seconds, quantitative comparisons of translocation vary due to the factors explained above. We maintain the comparison to electrical stimulation in the main figure because it represents a more reproducible experiment and shows better the translocation of proteins at the synapse.

In general, using field stimulation in pHluorin experiments enhances reproducibility in exocytosis studies and is considered the gold standard in the field. We have added a phrase to explain our choice of field stimulation when using pHluorin constructs in our work, as suggested (see page 6, first paragraph).

2. The authors claimed that the approach revealed all biotinylated proteins, which appeared as a smeared band spanning most molecular weights in cells expressing LRRTM-HRP construct (Figure 1F), and quantification of total biotinylation rates revealed no significant differences (Supp. Fig. S1C, D). Theoretically, KCl treatment causes more proteins translocated to synaptic cleft. The authors should provide evidence to show western blot of all biotinylated proteins from the isolation of synaptic cleft in the presence of KCl.

We have performed new experiments to quantify isolated biotinylated proteins from the synaptic cleft, as suggested. These results are now presented in the new Supp. Figure S2C, D. The experiments show similar results to those in the first version of the manuscript, which used total lysates to quantify biotinylation rates. This similarity is expected, given that biotinylation occurs only at the cleft when combining the specific localization of the enzyme with the use of impermeant variants of biotin phenol. Therefore, similar results are expected when measuring biotinylation from total lysates versus isolating biotinylated proteins and then measuring biotinylation rates, as both samples should contain the same biotinylated proteins. These new results are now mentioned on Page 4, first paragraph.

We do not observe increases in cleft biotinylation after KCl. There are some factors that may contribute to this result. First, the total amount of proteins exposed at the cleft after SV exocytosis may not be significant enough to increase the already dense proteome of the synaptic cleft, thus contributing only a relatively small percentage change that may be beyond detection. And second, synaptic vesicle exocytosis may drive simultaneous endocytosis of other surface proteins before being biotinylated, as could be seen during ultrafast endocytosis events, perhaps reducing the total number of cleft proteins during KCl and compensating for new proteins translocated, keeping the global proteome without changing significantly. We agree that this is an interesting point and added some of these ideas in the revised discussion (see Pag. 7, last paragraph).

3. In addition, the authors should perform LC-MS/MS to identify all biotinylated proteins from the isolation of synaptic cleft in the presence of KCl. Proteomic mapping of endogenous synaptic proteins in activity-driven trafficking would be a very useful resource. Please refer to Ting's work (Cell. 2016 Aug 25;166(5):1295-1307).

We agree on the value of this experiment. However, we believe this is out of the scope of the current manuscript. Future experiments directed towards scaling this technology for proteomic detection will be of great interest. We now add a phrase in the discussion to highlight this point (Pag. 8, second paragraph).

4. In this manuscript, the authors checked synaptic proteins during depolarization with KCl. I would expand the approach in excitatory synapses or inhibitory synapses.

We agree on the value of exploring the usability of this technique for inhibitory synapses. However, we believe this is out of the scope of the current manuscript.

5. In this approach, coordinating proximity labeling times and translocation dynamics is essential to detect relative changes in surface abundance of trafficking proteins. The authors claimed that 15s is optimal biotinylating time and translocation time. Proteins from different trafficking pathways vary, and they take different time to reach plasma membrane. So it is very different to capture most of endogenous translocation proteins in the complicated context of cellular biology.

We now add a phrase in the discussion to take this additional complexity into account: “We optimized our approach for detecting fast events occurring at synapses, but it is worth noticing that perhaps slower synaptic trafficking pathways could benefit from additional optimization by coordinating labeling times with trafficking properties.” See pag 8, first paragraph.

**Minor comments:**

1. Page 2, 3rd paragraph: HPR should be HRP. Fixed

2. Page 2, 3rd paragraph: H202 should be H2O2 Fixed

3. Page 21, the legend of Figure 1: "potassium (KCl)" should be "potassium chloride (KCl)". Fixed

4. Page 22, Figure 2A: the WB picture was cropped unproperly. Please provide full picture for the Syp1 in the Input. Fixed. Also uncropped blots for everything are shown in Supplementary Materials.

5. Glutamate Receptor 1 should be abbreviated as "GluR1" (Figure 2B and 2C) or "GluR I" (in the text)? Fixed

6. Please label the scale bar in Figure 3D. Noted in the figure legend.

**Reviewer #3:** Pascual-Claro and de Juan-Sanz report a new approach and use of the repurposed technology to evaluate the transient exposure of synaptic vesicle (SV) proteins to the extracellular milieu. The authors built on the proximity labelling approach coupled with elicited increased synaptic activity to promote exocytosis of numerous SVs, thus increasing the abundance of the (integral) SV proteins at the synaptic cleft. The use of the fast kinetics of the HRP enzyme allowed the authors to study the trafficking of endogenous proteins to the plasma membrane of the synapse. Although similar approaches to study these questions have been documented in the literature (as noted by the authors too), this field would benefit from an unbiased proteomic-based approach to identify proteins present in the synaptic membranes shortly after stimulation.

Yet the novel scientific outcomes gained from this technology are somewhat underwhelming, based on the presented examples of the neuronal pentraxin 1 (a soluble, secreted glycoprotein) and Autophagy-Related 9A (ATG9A). Given that the findings are presented through the methodology-driven manuscript, it is not clear if the identification of labelled proteins is still undergoing. Either way, there are several points that should receive further attention:

1. The authors state that they used LRRTM1-HRP and LRRTM2-HRP to perform proximity labelling. However, it is not clear which construct is used in which experimental setup. This should be clarified in the manuscript.

We now emphasize in the text that we use both constructs simultaneously (see page 3, last paragraph). We have also detailed this further in the main text methods section (page 12, first paragraph). Additionally, we have added a new extended supplementary protocol that provides a detailed procedure to facilitate the reproducibility of the presented method, including a thorough description of the use of both constructs.

2. It may be useful to detail how synaptic cleft isolation is performed here. Do the results reveal biotinylated proteins present at the pre-synapse after de-enrichment of post-synaptic densities? The list of biotinylated proteins after stimulation (or pre/post stimulation difference) by mass spectrometry, and verified by Western blotting for key examples, would be useful.

We have added all details of the synaptic cleft isolation procedure in a new extended supplementary protocol to clarify this point. Briefly, our method isolates the entire synaptic cleft as done before by the Ting Lab (Loh et al, 2016; containing both pre- and post-synaptic proteins). Given the known physiology of exocytosis events at the presynapse, we use this method to quantify the translocation of endogenous presynaptic proteins, although this method should potentially be able to quantify postsynaptic exocytosis (if one knows a protein translocated to the postsynaptic surface during 15 seconds of depolarization). We added a new phrase in the discussion to clarify this point (see page 7, beginning of last paragraph).

We agree on the value of characterizing the activity-driven exocytosis proteome. However, we believe such a complicated experiment is out of the scope of the current manuscript. Future experiments directed towards scaling this technology for proteomic detection will be of great interest and thus we now add a phrase in the discussion to highlight the importance of this future development (Page 8, second paragraph).

3. Although LRRTM-HRP is expressed and only active when H2O2 and biotin are added (which should label only the surface-exposed proteins), the enzyme can be activated intracellularly at the time when H202 is added to the culture. Although the authors used an impermeant version of biotin, biotin is usually present in the culture media and can be available to the HRP ligase that is being trafficked intracellularly. Is any sort of biotin starvation performed prior to the KCl/biotin administration? Also, is there any biotinylation in the post-synaptic cell during the 15s stimulation? This can be addressed by biotin staining and/or by evaluating the presence of intracellular post-synaptic proteins after biotin pull-down. Specifically, Fig 2. panel A: is PSD-95 present in the isolated fractions? The authors should include the Western blot showing enrichment for biotin.

We do not perform biotin starvation, which we now emphasize in the new extended supplementary protocol for clarifying this point. The surface of the postsynaptic site will indeed be biotinylated during the 15s stimulation - we aimed to show that in the diagram of Fig. 1A (both left and right panels), in which we depicted both pre- and post-synaptic biotinylation by adding red “biotinylation dots'' in proteins present at pre- and post-synapse. We added a phrase in figure legend for 1A to clarify this point (“Biotinylation occurs at pre- and post-synaptic surfaces…”; see pag. 21, Figure 1A legend) and we now add a legend for Fig 1A, which should help to make clear that the postsynaptic surface is biotinylated.

Our protocol builds up on careful extensive validation done previously to disassemble possible detergent-insoluble postsynaptic structures (including adding a 1% SDS lysis step with 10 min boiling to disrupt the postsynaptic density, as described by Loh et al 2016 and shown in our materials and methods). However, we now highlight this also in the text to reassure the reader that experiments are done in these conditions (see pag. 4, last part of third paragraph).

To further confirm that biotinylation occurs exclusively extracellularly when combining LRRTM1/2 and BxxP as previously described by Loh et al. 2016, we performed biotin staining using Streptavidn-Alexa 647 in neurons expressing LRRTM1/2-HRP or cytosolic APEX in the presence of BxxP and H_2_O_2_. As expected, 15s biotinylation driven by BxxP only occurred when the enzyme was accessible extracellularly, showing that no significant biotinylation occurs inside neurons with this approach (see Supp. Figure S2E). This supports the initial observation that cytosolic proteins are not part of the synaptic cleft sample, as shown by western blots against SNAP25 or Rab3, two cytosolic proteins abundant in synapses. This experiment is now also discussed in the results section, page 5, first paragraph.

To evaluate the extent to which PSD-95 could be isolated in our synaptic cleft enrichment conditions, we loaded new gels with samples from the experiment shown in Fig 2A. While we robustly detected PSD-95 in the input, we could not detect isolation of PSD95 in synaptic cleft preparations. This new result in Fig. 2A is now discussed in the results section (see Pag. 4, third paragraph) and confirms that we can reproduce previous results described in Loh et al Cell 2016.

4. Fig 2.: SNAP-25 may not be ideal control in this context - it may be good to include other/additional control protein.

SNAP25 is highly abundant in synaptic vesicles, whose translocation is shown here by quantification of vGlut1 and Syp1 (and also Syt1 in the revised manuscript). We believe this is a good control for testing whether there is intracellular biotinylation in our conditions. However, as suggested, we have also confirmed that the cytosolic protein Rab3 is not isolated in synaptic cleft samples, yet its abundance is clearly observed by western blot in the total lysate. We now include this additional control in Fig. 2A and the result is discussed in the results section (see Page 4, 4th paragraph).

5. Fig 2. panel D: usually very little vGlut1 is present at the neuronal surface under the resting conditions (here control/C). It is surprising that much vGlut1 is detected after total surface isolation of biotinylated proteins. This control experiment, and the use of vGlut1 as an example here, is confusing and may not be ideal to convey the point that the authors wish to make.

We realize that in the pictures shown in Fig. 2D, the band corresponding to the input was saturated for the chosen exposure time for all three antibodies. We have updated the figure to correct this, showing the same experiment as before but with less exposure. Now a less intense band in the TM isolation proteins appears when compared to the input.

Indeed previous estimates show that ~3% of vGlut1 is located on the neuronal surface (Kim and Ryan, 2009, J Neurosci). The reason why we can detect such a small fraction in respect to the input is because the input represents a much smaller fraction in terms of protein loaded versus the amount of protein from which the enrichment is performed. In this case, the input line corresponds to 1% (5𝜇g) of the amount of protein used for surface isolation (500𝜇g). This was necessary to be able to detect synaptic proteins in samples corresponding to total surface-enriched samples to be able to explore whether HRP-TM could be used for capturing synaptic translocation of proteins. Thus, despite only 3/100 vGlut molecules could be potentially detected from the surface, we force their detection in the western blot by using 100x more protein as the initial sample for isolation compared to the input. Although one cannot expect 100% efficiency in the retrieval of that 3% vGlut surface, the small amount that is isolated becomes detectable when using 100 times more protein for isolation. This allows us to see in a single blot both samples under the same exposure conditions. Of note, we typically use 100𝜇g as a sample for isolation of synaptic cleft proteins (instead of 500𝜇g here), which also shows that surface TM labeling is inadequate for isolating synaptic proteins, in agreement with the expectations mentioned by the reviewer.

To clarify this important point, we now 1) show how much the input corresponds to in comparison to the sample used for isolation throughout all the figures of the manuscript, 2) add in figure legends the amounts of protein used in each case and 3) mention an explanation in the results section to clarify this point (see page 5, second paragraph).

6. Elevated extracellular potassium chloride is commonly used to achieve membrane depolarization of cultured neurons, yet it does not always induce neuronal activity and it is considered to be rather poor representation of in vivo phenomena. Does electrical stimulation of cultured neurons yield the same results?

The problem of using electrical stimulation in combination with proximity labeling would be very interesting to solve. However, stimulating millions of neurons simultaneously for cleft isolation using field stimulation is not trivial, i.e. electrical stimulation of the large plates of cultured neurons used here poses significant challenges due to the i) high resistance of the medium given the large distance between the electrodes and ii) the difficulty in evenly distributing the electric field in rounded plates. In the current system, the high currents required for neuronal depolarization will also lead to significant heating of the sample and uneven depolarization, likely reducing reproducibility. Future work should focus on developing specialized rectangular culture plates to 1) evenly distribute the electric field and 2) reduce the distance between the customized long electrodes spanning the two sides of the dish. Or perhaps an easier alternative will be to combine optogenetic stimulation of the entire culture and proximity labeling. While very interesting, we believe this extensive optimization work is out of the scope of the current manuscript. We now mention this possible future optimizations to avoid using KCl in the future (see Pag. 7, last paragraph). It is in any case perhaps worth mentioning here that fifteen seconds of KCl cause synaptic vesicle exocytosis to levels not so different from those obtained during fifteen seconds of electrical stimulation at 10Hz (Figure 1, using synaptophysin-pHluorin).

7. Given the nice and inclusive introduction, it may be good to also cite the work presented in 10.1016/j.cell.2023.05.044

We added this citation in the introduction when discussing proximity biotinylation (see pag. 2, third paragraph).

8. I appreciate the indicated (minor) gap in knowledge regarding neuronal pentraxin 1, yet it may be useful to elaborate why, out of all possible options and given limitations of this technology, the attention has been drawn to a soluble secreted protein. Are many other soluble proteins labelled too?

We recently found that activity drives the surface translocation of another soluble synaptic organizer, LGI1 (Cuhadar et al 2024, Cell Reports). We were curious to see if that would be true for other proteins and given the literature available we explored activity-driven translocation of NPTX1, aiming to also provide novel biological data for this manuscript for a broader readership. We agree that it will be interesting in the future to see to what extent other soluble synaptic organizers can be identified to be translocated. We added a phrase in the results to elaborate on this point (see page 6, first paragraph).
