# Supplementary material for: Monitoring of activity-driven trafficking of endogenous synaptic proteins through proximity labeling
Source: PLoS Biol. 2024 Oct 28;22(10):e3002860. doi: 10.1371/journal.pbio.3002860 (PMC11542813; doi:10.1371/journal.pbio.3002860)
Supplement: Attachment — Submitted filename: PLoS Biology revision comments R2.docx [file pbio.3002860.s009.docx]

We thank reviewers and editors for the time spent to revise our manuscript. The point-by-point response to the reviewers and explanations related to their comments are listed below:

**Editorial changes:**

Please also make sure to address the following data and other policy-related requests:

* We would like to suggest a different title to improve readability/accuracy: "Monitoring of activity-driven trafficking of endogenous synaptic proteins through proximity labeling" - Fixed. We agree this improves readability/accuracy.

* Please add the links to the funding agencies in the Financial Disclosure statement in the manuscript details – Fixed

* Please include the approval/license number of the ethical approval for the animal experiments. - Fixed

**Reviewer #1:** We thank the authors for addressing most of our concerns. Our only remaining commentary regards the Supplementary Figure 1. We have two comments:

1. There seems to be some typos and mismatches between figure correspondence in the Table and the Figures of the paper, including references to statistically significant differences in the graphs of the main Figure, which do not apear as significant in the table (eg comparison between 15 and 30s has a p-value of 0.086 that is reported as significant in the main Figure). Adding p-values to the main figure legends will help this too.

We sincerely thank the reviewer for identifying the mismatches between the figures and the supplementary table. Upon a thorough review, we found that only Figure 2B contained discrepancies, which have now been corrected. We have ensured that all references to statistical significance in the figures and table are consistent.

Regarding the suggestion to add p-values to the figure legends, we believe that this would make the figure legends less readable, as the supplementary table already provides detailed statistical information. We would prefer to keep the legends concise to maintain clarity. However, we now refer in the legends to the supp. statistics table for clarity, stating that “Table S1 shows the number of experiments and replicates, means and error, and statistical tests used.”

2. We also have a question about the statistical methods used. The ratios, proportions or percentages values analyzed by the authors correspond to categorical variables, not numerical values. If so, they should be compared using a Chi-square test rather than a t-test or ANOVA test.,

We thank the reviewer for their comment regarding the choice of statistical methods. We would like to clarify that while our data includes ratios, proportions, and percentages, these variables are continuous numerical variables, not categorical variables.

A categorical variable represents distinct categories or groups without a natural numerical order (e.g., gender, treatment group) and is typically analyzed using non-parametric methods like the Chi-square test as noted. In contrast, our variables are continuous, representing a range of values with meaningful numeric properties that can be ordered and compared mathematically. As such, parametric and non-parametric tests such as t-tests or ANOVA are suitable for analyzing these data, allowing for comparison of means or ranks across different groups.

We have consulted with biostatistics colleagues, who concur that the statistical methods we used are appropriate for our data. Based on this clarification, we believe the current analyses are correct and do not require changes. We hope this addresses the reviewer's concern and are happy to provide any additional clarification if needed.

**Reviewer #3:** I appreciate the efforts that authors put in the revision of original manuscripts, yet those efforts were predominantly focused on clarifications of the original work and adding additional controls. I was not aware of authors recent Cell Reports paper when originally reviewing this work (not published at that time point, I think). The suggested experiment that authors consider to be outside the scope of this work would, in my view, make this publication stronger and add to the novelty.

This manuscript contains a nice set of data, which is now made even stronger through the revised work, yet the novelty is still limited. A good opportunity has possibly been missed here.

That said, I support publication of dataset presented here.

We thank the reviewer for appreciating our revisions and supporting the publication. While we understand the suggestion for an additional experiment to enhance novelty, we still believe it is beyond the scope of the current manuscript but will consider it for future work of our lab.
